# Complementary and alternative medicines for behavioral and psychological symptoms of dementia: A protocol of overview of systematic reviews

**DOI:** 10.1097/MD.0000000000026397

**Published:** 2021-06-25

**Authors:** Chan-Young Kwon, Boram Lee

**Affiliations:** a Department of Oriental Neuropsychiatry, Dong-eui University College of Korean Medicine, Busan; b Department of Clinical Korean Medicine, Graduate School, Kyung Hee University, Seoul, Republic of Korea.

**Keywords:** BPSD, complementary and alternative medicine, dementia, integrative medicine, overview

## Abstract

**Background:**

Dementia is causing a huge medical and socioeconomic burden. Along with strategies to delay cognitive decline in dementia, behavioral and psychological symptoms of dementia (BPSD) are major contributing factor to the burden of dementia, and have been an important clinical issue for successful management of dementia. However, pharmacological strategies such as antipsychotics raise concerns in terms of risk-to-benefit ratio in managing BPSD. Therefore, there is a need for an effective and safe alternative in BPSD management. From this point of view, various complementary and alternative medicines (CAMs) are attracting attention in BPSD management. Therefore, the overview will make it possible to evaluate the feasibility of using CAM as a potential treatment strategy for BPSD in terms of evidence-based medicine.

**Methods and analysis:**

Comprehensive searching will be performed in 13 bibliographic databases from their inception dates to November 2021. Systematic reviews and/or meta-analyses that examined the effectiveness and safety of CAM modalities including herbal medicine, acupuncture, acupressure, aromatherapy, meditation, and relaxation on BPSD, will be included. The methodological quality of included reviews will be assessed by using the A MeaSurement Tool to Assess systematic Reviews-2. Two independent researchers will conduct study search, study selection, data extraction, and quality assessment processes.

**Results:**

The results of overview will be disseminated by the publication of a manuscript in a peer-reviewed journal or presentation at a relevant conference.

**Conclusion:**

The findings of this overview will help to solve the major public health problem related to dementia, and will provide patients with dementia, their caregivers, clinicians, and health policy makers credible evidence in mitigating the burden of dementia.

**Ethics and dissemination:**

As this protocol is for an overview of systematic reviews and meta-analyses, ethical approval is not required.

**Protocol registration number:**

Open Science Framework registry (https://osf.io/g5f3m)

## 1 Introduction

Owing to the increase in the aging population worldwide, dementia has become a significant medical and socioeconomic burden.^[1,2]^ By 2040, 81.1 million individuals are estimated to be affected by dementia.^[3]^ Since 2003, no antidementia drugs have been approved by the United States Food and Drug Administration for the treatment of Alzheimer disease (AD), a representative type of dementia, and the development of anti-dementia drugs has been unsuccessful.^[4]^ Based on previous unsuccessful clinical trials, some challenges, such as early intervention in AD progression, exploration of effective drug doses, exploration of AD pathology-specific therapeutic targets, and understanding the complex pathophysiology of AD, have been identified, and research in this field is advancing.^[4]^

In addition to strategies to delay cognitive decline associated with dementia, behavioral and psychological symptoms of dementia (BPSDs) have been an important clinical issue for the successful management of dementia and relieving the medical and socioeconomic burden of dementia.^[5]^ Although the characteristic BPSDs may differ according to the dementia type, most patients with dementia experience 1 or more BPSD (s) regardless of the dementia type.^[6]^ An individualized and comprehensive approach is emphasized for the effective management of BPSDs, and nonpharmacological approaches are recommended first.^[7,8]^ However, pharmacological strategies such as antipsychotics raise concerns in terms of the risk–benefit ratio in managing BPSDs.^[7]^

From this perspective, various complementary and alternative medicines (CAMs) have attracted attention for BPSD management.^[9]^ For example, CAM modalities such as aromatherapy and acupressure have been shown to be effective in improving agitation in patients with dementia,^[10]^ suggesting that CAM has the potential to provide a safe and effective strategy for BPSD management. An overview of systematic reviews, also called an umbrella review, helps to comprehensively review a very wide range of topics by reviewing previously published relevant systematic reviews and/or meta-analyses and has the potential to provide the highest quality of evidence.^[11]^

Therefore, an overview of systematic reviews may be a suitable research methodology to comprehensively evaluate the level of evidence for the effectiveness and safety of various types of CAM modalities for managing BPSDs and critically review the existing clinical evidence. However, no umbrella review has thus far comprehensively reviewed various types of CAM modalities for BPSD management. The purpose of this protocol is to provide an overview of existing systematic reviews and/or meta-analyses reporting the effectiveness and safety of CAM modalities, including herbal medicine, acupuncture, acupressure, aromatherapy, meditation, and relaxation, for BPSD management. The results of this overview will make it possible to evaluate the feasibility of using CAM as a potential treatment strategy for BPSDs in terms of evidence-based medicine, which will contribute to the resolution of the dementia epidemic that has a significant medical and socioeconomic burden globally.

## 2 Methods

### 2.1 Study registration

The systematic review protocol is registered at the Open Science Framework registry (osf.io/46nzm). If protocol amendments occur, the dates, changes, and rationales will be tracked in the registries. This protocol is in accordance with the Preferred Reporting Items for Systematic Review and Meta-Analysis Protocols 2015 statement^[12]^ (Supplement 1).

### 2.2 Data sources and search strategy

A comprehensive search will be performed in the following electronic bibliographic databases from their inception dates to November 2021: 6 English databases (MEDLINE via PubMed, EMBASE via Elsevier, Cochrane Central Register of Controlled Trials, Allied and Complementary Medicine Database via Elton B. Stephens COmpany, Cumulative Index to Nursing and Allied Health Literature via Elton B. Stephens COmpany, and PsycARTICLES via ProQuest), 2 Chinese databases (China National Knowledge Infrastructure and Wanfang Data), and 5 Korean databases (Oriental Medicine Advanced Searching Integrated System, Koreanstudies Information Service System, Research Information Service System, Korean Medical Database, and Korea Citation Index). Moreover, to identify additional gray literature such as thesis or conference abstract for inclusion, the reference lists of the relevant papers will be reviewed, and a manual search on Google Scholar will be performed. Two independent researchers (C-YK and BL) will perform the study search process. The search strategy for MEDLINE via PubMed is presented in Table 1.

**Table 1:** Search strategies for the Medline via PubMed.

#1. Dementia[Mesh] OR dement∗[tiab] OR Alzheimer∗[tiab] OR “Lewy body”[tiab] OR Huntington∗[tiab] OR Parkinson∗[tiab] OR “Pick disease”[tiab] OR “cognitive impairment”[tiab]
#2. “Herbal Medicine”[Mesh] OR “Drugs, Chinese Herbal”[Mesh] OR “Medicine, East Asian Traditional”[Mesh] OR herb∗[tiab] OR Acupuncture[Mesh] OR “Acupuncture Therapy”[Mesh] OR “Acupuncture Points”[Mesh] OR acupunct∗[tiab] OR Acupressure[Mesh] OR acupressure[tiab] OR Aromatherapy[Mesh] OR aroma∗[tiab] OR Meditation[Mesh] OR meditation[tiab] OR Relaxation[Mesh] OR “Relaxation Therapy”[Mesh] OR relaxation∗[tiab]
#3. “systematic review”[tiab] OR “meta analysis”[tiab] OR “meta-analysis”[tiab] OR “systematic review”[PT] OR “Systematic Reviews as Topic”[Mesh] OR “meta-analysis”[pt] OR “Meta-Analysis as Topic”[Mesh]
#4. #1 AND #2 AND #3

### 2.3 Inclusion criteria

#### 2.3.1 Types of studies

Systematic reviews and/or meta-analyses examining the effectiveness and safety of CAM modalities for BPSDs will be included. There will be no restrictions on the publication language.

#### 2.3.2 Types of participants

Systematic reviews and/or meta-analyses of original clinical studies involving individuals with any type of dementia in long-term care facilities, communities, or specialized geriatric assessment and psychogeriatric units will be included. There will be no restrictions on the sex, age, or race/ethnicity of the participants.

#### 2.3.3 Types of interventions

Systematic reviews and/or meta-analyses of original clinical studies involving any type of CAM modality as monotherapy or adjunctive therapies to psychotropic drugs such as anxiolytics, antidepressants, and antipsychotics with or without routine care for dementia as experimental interventions will be included. In this overview, the following CAM modalities alone or in combination with a comparator intervention will be included: herbal medicine, acupuncture, acupressure, aromatherapy, meditation, and relaxation. Regarding the comparator intervention, systematic reviews involving wait-list, placebo, or psychotropic drugs such as anxiolytics, antidepressants, and antipsychotics with or without routine care for dementia as control interventions will be included.

#### 2.3.4 Types of outcome measures

The primary outcomes will include the following validated measures of BPSDs such as Neuropsychiatric Inventory,^[13]^ Cohen–Mansfield Agitation Inventory,^[14]^ and Brief Psychiatric Rating Scale.^[15]^ The secondary outcomes will include the following: total effectiveness rate for BPSDs; activity of daily living (ADL) assessment of patients with dementia, such as the Barthel index^[16]^ and Katz index;^[17]^ instrumental ADL assessment of patients with dementia, such as ADL Prevention Instrument^[18]^ and Alzheimer Disease ADL International Scale;^[19]^ caregiver burden such as Caregiver Burden Inventory;^[20]^ quality of life of caregivers such as Short Form 36 Health Survey;^[21]^ placement in long-term care facilities from home; incidence of adverse events; and treatment discontinuation due to total or serious adverse events. Systematic reviews and/or meta-analyses that do not address the primary outcomes will be excluded.

### 2.4 Study selection

**Figure 1:**
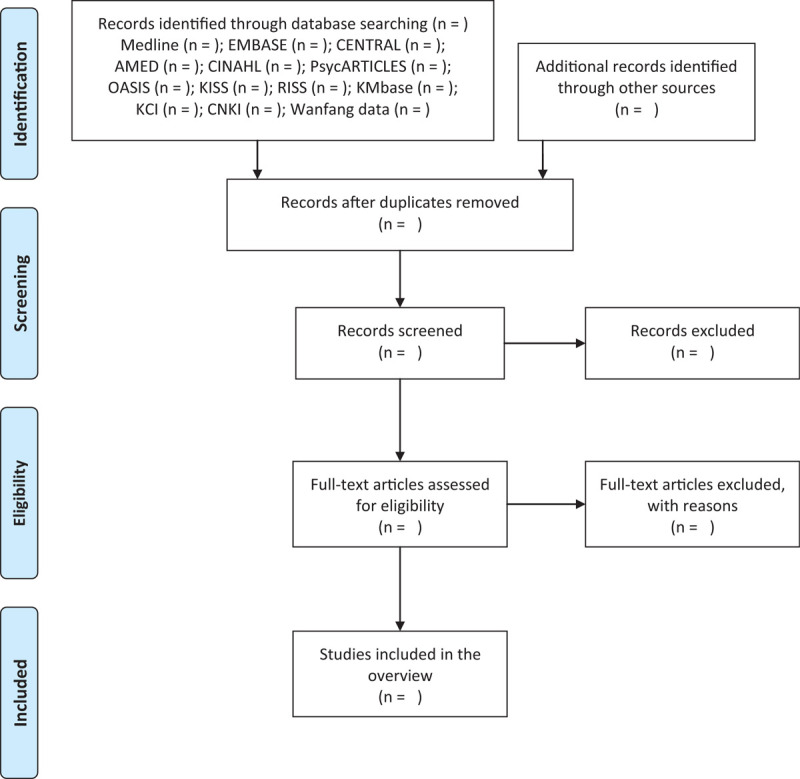
A PRISMA flow diagram of the literature screening and selection processes. AMED = Allied and Complementary Medicine Database, CENTRAL = Cochrane Central Register of Controlled Trials, CINAHL = Cumulative Index to Nursing and Allied Health Literature, CNKI = China National Knowledge Infrastructure, KCI = Korea Citation Index, KISS = Koreanstudies Information Service System, OASIS = Oriental Medicine Advanced Searching Integrated System, RISS = Research Information Service System.

First, the titles and abstracts of the initially searched studies after removing duplicates will be screened. The full texts of these potentially eligible studies will then be carefully reviewed for eligibility. The 2-step study selection process will be performed by 2 independent researchers (CYK and BL), and any disagreements between them will be resolved through discussion. To manage bibliography from the included articles, EndNote X8 (Clarivate Analytics, Philadelphia, PA) will be used. The study selection process will be described in PRISMA flow diagram (Fig. 1).

### 2.5 Data extraction

From the included systematic reviews and/or meta-analyses, 2 independent researchers (C-YK and BL) will use a standardized, predefined, pilot-tested data extraction form in Microsoft Excel 2016 to extract relevant data for the assessment of study quality and data analysis. The following information will be extracted: the first author's name, year of publication, country in which the study was conducted, number of original studies included, total sample size, search period, study design, details of the participants, interventions of the treatment group and control group, main outcome measures, results of the meta-analysis, the author's conclusion, safety data, and information for A MeaSurement Tool to Assess systematic Reviews (AMSTAR)-2. Any discrepancies in the data extraction process will be resolved through discussion by the 2 researchers. In this data extraction process, Microsoft Excel 2016 (Microsoft, Redmond, WA) will be used to perform the data extraction process.

### 2.6 Quality assessment

For the included systematic reviews and/or meta-analyses, the AMSTAR-2 tool will be used to assess the methodological quality.^[22]^ AMSTAR-2 is a validated critical appraisal tool for systematic reviews with 16 items, and all the items will be evaluated and rated as “yes,” “partially yes,” or “no.”^[22]^ Using this tool, by referencing the critical weaknesses and flaws of each systematic review and/or meta-analysis, the overall quality can be assessed and classified as “high,” “moderate,” “low,” or “critically low.”^[22]^ The quality assessment process will be performed by 2 independent researchers (C-YK and BL), and any disagreements between them will be resolved through discussion. In addition, Microsoft Excel 2016 (Microsoft, Redmond, WA) will be used during this process to perform quality assessment.

### 2.7 Data synthesis and analysis

A narrative (descriptive) synthesis of all included systematic reviews and/or meta-analyses is planned. The data obtained from the included studies will be presented as odds ratios or risk ratios for dichotomous data and mean differences or standardized mean differences for continuous data, with their 95% confidence intervals.

### 2.8 Ethics and dissemination

As this protocol will provide an overview of systematic reviews, ethical approval is not required. The results of the overview will be disseminated by the publication of a manuscript in a peer-reviewed journal or presentation at a relevant conference.

## 3 Discussion

The medical and socioeconomic burden of dementia is increasing worldwide, and priority efforts are needed to alleviate this burden.^[1]^ Management of BPSDs, a major factor in caregiver burden, is a realistic management strategy for dementia as disease-modifying drugs for dementia have not been developed thus far.^[5]^ Given the limitations of conventional pharmacological approaches for managing BPSDs,^[7,8]^ an effective and safe alternative is urgently needed, and CAM modalities may be a breakthrough to improve BPSD management. However, prior to being officially recommended, CAM modalities must be critically evaluated in terms of their effectiveness and safety in the context of evidence-based medicine, and this umbrella review will help elucidate the strengths and weaknesses of the evidence of various CAM modalities for BPSD management. Therefore, we believe that the findings of this overview will help solve the major public health problems related to dementia and provide patients with dementia, their caregivers, clinicians, and health policy makers credible evidence in mitigating the burden of dementia.

## Author contributions

**Conceptualization:** Chan-Young Kwon.

**Funding acquisition:** Chan-Young Kwon.

**Methodology:** Chan-Young Kwon, Boram Lee.

**Supervision:** Chan-Young Kwon.

**Writing – original draft:** Chan-Young Kwon.

**Writing – review & editing:** Chan-Young Kwon, Boram Lee.

## Supplementary Material

Supplemental Digital Content
